# A Nurse's Charter of Liberty

**Published:** 1918-05-25

**Authors:** 


### THE ECONOMICS OF NURSING.

### A Nurse's Charter of Liberty.

As I pointed out in a previous article, the word "emolument" is comprehensive, and does not signify merely financial reward. Man doth not live by bread alone! I propose on this occasion to discuss the working hours of hospital nurses, and in this connection I wish to express my indebtedness to Lord Knutsford, the distinguished Chairman of the London Hospital, who has been good enough to favour me with an illustrated pamphlet, of which he is the author, entitled "Nursing as a Profession." This pamphlet relates chiefly, but not exclusively, to the London Hospital, and its mission is to offer advice and warning to intending candidates.

The London Hospital possesses the unique distinction of being the largest nursing institution in the kingdom, and in point of population it rivals many a little town with pretensions to importance. Its reputation is world-wide, and nurses who have graduated there occupy some of the highest and most honourable positions in the profession, including the Navy and the Army—including also not only the British Empire, but every civilised country in both hemispheres. It is in the full sense a truly great institution, worthy alike of the Metropolis and of the spirit that animates the British people. From the probationer's standpoint, as hospitals and hospital service go, the London Hospital is ideal. There is no entrance fee: a progressive salary is paid from the outset; the training is first class, and a pension is awarded at the age of forty-five. The majority of hospitals require a three years' period of training. The London Hospital authorities regard two as sufficient, with a proviso that there shall be two further years of compulsory service. The present is not an opportune moment for discussing this particular feature of training. It is a subject concerning which there is much diversity of opinion. But I may point out in passing that, bearing in mind the limited professional life which the average working nurse enjoys, if efficiency can be secured with two years of training this standard should be made general.

The feature of the pamphlet which most interests me is what the noble author describes as "the nurse's Charter of Liberty." Here is the complete text: "No woman ought to enter any hospital where she gets less than two hours off duty every day in daylight, half a day off every week, one whole day off every month, two or three weeks' holiday in the year, or the equivalent of these hours differently arranged." Any Charter of Liberty immediately and of necessity creates an *arrière pensée* of days of bondage, and therefore when I saw the title I expected great things. The hard-worked nurse, I thought, had surely found a champion, and the hour of her emancipation had arrived. Need I say that I was bitterly disappointed! The text of the Charter is the anti-climax of its title. It is more: it is a veritable caricature. But lest peradventure that my judgment may be warped, or my vision oblique, I should like to ask Lord Knutsford to amplify his Charter of Liberty, and to explain on what human principle its provisions are based.

The supreme emolument of the worker, and especially of the woman worker, is freedom. It is the priceless privilege for which the whole civilised world is now pouring forth its blood and treasure, and beside which, in comparison, money does not count, and therefore I take the liberty of asking Lord Knutsford to state in precise terms exactly why a nurse should be called upon above all other workers to forfeit her freedom. Why should she regard herself as possessing a Charter of Liberty if she is permitted to enjoy one day off duty every month instead of every week, and two hours of daylight leave when in other walks of life the workers enjoy quite three times this amount? Lord Knutsford iterates and reiterates that "nursing is a hard life." Why should it be a hard life? A nurse is not a religious recluse, but a woman worker engaged at womanly work, and out for a career. May I be permitted to suggest that I do not think Lord Knutsford quite appreciates this? His tone, despite such high-sounding words as a Charter of Liberty, is the tone of King John. "There is a growing tendency on the part of nurses in every hospital," he says, "as the conditions of service are improved, to demand as a right—and to growl if they do not get them—privileges which but a few years back were never dreamt of." I am glad to have his lordship's testimony as to this. I rejoice in the growing tendency to growl!

Just examine the actual facts. Take the case of a young woman brought up in a refined home beginning her career in a hospital situated in the heart of Whitechapel. She is to thank the gods if she is permitted to wander round the purlieus of that delectable neighbourhood for two hours every day. If she took a 'bus West she would scarcely have time to shake hands with the lions in Trafalgar Square before it was time for her to return. When all the world and his wife turn out on Saturday afternoon she must be at her post. According to the liberal programme of the London Hospital, "she will have four hours off on Saturday, and on Sunday she is allowed to attend any religious service she likes." To my untutored mind these conditions must have found their origin in Dartmoor. Lord Knutsford presses home on the would-be nurse the necessity for sacrifice. He demands her body and her soul. He does not give her credit for ambition, nor admit her claim to a career. He writes, so he says, for women who have every good quality that a nurse should have *wishing to earn their livelihood.* His lordship provides a livelihood, but nothing more. The sweetness and the joy of liberty exist for the nurse only in the title of their Charter.

Now what is the underlying motive for this sweating? Is it moral, religious, or financial? I have searched the pages of Lord Knutsford's pamphlet for an answer, but my search is vain. I can find no explanation why a nurse in the great London Hospital should not enjoy four hours off duty every day, a half-day every week, and in addition one day's rest in seven. Perhaps the distinguished author will provide a solution to the riddle.

